# Ichneumonidae species (Hymenoptera) new to Iraq recorded from Kurdistan

**DOI:** 10.3897/BDJ.13.e161156

**Published:** 2025-07-18

**Authors:** Srwa Kareem Hamad

**Affiliations:** 1 Department of Plant Protection, College of Agricultural Engineering Sciences, Salahaddin University, Erbil, Iraq Department of Plant Protection, College of Agricultural Engineering Sciences, Salahaddin University Erbil Iraq

**Keywords:** new records, taxonomy, identification, Ichneumonoidea, parasitoids

## Abstract

**Background:**

Ichneumonidae, a dominant and ecologically important family of parasitoids in the large insect order Hymenoptera, are poorly studied in Iraq, with only 18 previously recorded and identified species known so far. During a survey in Kurdistan, Iraq, numerous Ichneumonidae species were collected in July 2023 using a Malaise trap.

**New information:**

Fourteen species of Hymenoptera: Ichneumonidae are herein newly recorded for the fauna of Iraq: *Phaenolobushilalii* Kolarov & Gürbüz, 2010; *Lissonotadeversor* Gravenhorst, 1829; *Alcimaorbitale* (Gravenhorst, 1829); *Campoletisscyticus* Riedel, 2017; *Casinariatenuiventris* (Gravenhorst, 1829); *Cryptusspinosus* Gravenhorst, 1829; *Perilissusdissimilator* Aubert, 1987; *Diadromuscollaris* (Gravenhorst, 1829); *Clistopygaincitator* (Fabricius, 1793); *Itoplectistunetana* (Schmiedeknecht, 1914); *Pimplaflavicoxis* Thomson, 1877; *Pimplaspuria* Gravenhorst, 1829; *Zaglyptusmulticolor* (Gravenhorst, 1829) and *Zatypotabohemani* (Holmgren, 1860). Recorded distributions are provided for all species, summarised for very widely distributed species.

## Introduction

Hymenoptera, one of the large insect orders (Stevens et al. 2007), is dominated by many groups of specialised parasitoids, of considerable importance in most terrestrial ecosystems (Polaszek and Vilhelmsen 2023). The family Ichneumonidae is the largest of these comprising, when last catalogued (Yu et al. 2016) 1.601 genera and 25,285 species. Hosts include Coleoptera, Hymenoptera and Lepidoptera larvae and pupae, as well as other arthropod groups (Broad et al. 2018, Çoruch and Kolarov 2010).

Ichneumonidae are considered potential suppressors of populations of pest species and are widely used in biological control programmes and natural enemy conservation efforts (Gupta 1991). However, despite their abundance, ecological and economic importance, ichneumonid biology remains relatively poorly studied (Shaw and Hochberg 2001). The Iraq fauna is especially poorly known, with only 18 previously recorded and identified species so far formally recorded from the country (Aljaafari 2022, Kareem et al. 2020, Kareem et al. 2024, Yu et al. 2016).This study is based upon material of the family Ichneumonidae collected from a single locality in Kurdistan in Iraq. The purpose of the study was to contribute to the knowledge of ichneumonid species in Iraq.

## Materials and methods

Specimens were collected using a Malaise trap from April to June 2023 at various agricultural areas in the Kurdistan region, which is located in the north of Iraq. All species recorded herein as new to Iraq were from the same locality and date: IRAQ: Kurdistan, Erbil Governorate, Qala Sngi Khwaru, 36°20'06.0"N 44°19'37.2"E 28.v.2023 (note that this locality has previously been spelt as follows: “Qal ah sanj”). Specimens were collected into 96% ethanol and preserved following Eastop and van Emden (1972) for later identification and transported to NHMUK London. They were mounted on card points and provisionally identified by Sir Anthony Galsworthy, a Scientific Associate at the Natural History Museum (NHMUK). Identifications were then confirmed by Dr Gavin Broad, Ichneumonoidea curator at NHMUK. Selected specimens have been imaged at NHMUK using a Hirox HRX-01 digital microscope (Hirox Co. Ltd., Tokyo, Japan) with software S/N H3265 Version 2.24, including built-in stacking software. All specimens are deposited at NHMUK.

## Checklists

### Ichneumonidae species new to Iraq

#### Acaenitinae

Förster, 1869

BA3C2740-D5F1-53B8-ABA2-F8CC7B083D9F

#### Phaenolobus hilalii

Kolarov & Gürbüz, 2010

4C936698-6642-5DC2-A0A8-58C058FDD4CD

https://www.gbif.org/occurrence/5084974714

##### Materials

- **Type status:** Other material. **Occurrence:** occurrenceDetails: http://api.gbif.org/v1/occurrence/search?occurrenceId=247781d3-455f-4b95-9ff7-cbcd88711e08; catalogNumber: NHMUK010888495; recordedBy: [Dr Srwa Karim Bandyan]; individualCount: 1; sex: Male; lifeStage: Adult; occurrenceStatus: PRESENTE; otherCatalogNumbers: NHMUK:ecatalogue:11380974; occurrenceID: 9D197442-6DA8-5E4B-B7F3-55064241AB30; **Taxon:** higherClassification: Animalia; Arthropoda; Insecta; Hymenoptera; Ichneumonoidea; Ichneumonidae; Acaenitinae; **Location:** locality: Kurdistan, Qala Snigi Khwaru; decimalLatitude: 30.335; decimalLongitude: 44.327; geodeticDatum: WGS84; **Identification:** identifiedBy: Dr Gavin R. Broad; **Event:** samplingProtocol: Malaise trap; eventDate: 2023-05-28; **Record Level:** institutionCode: NHMUK; collectionCode: BMNH(E); basisOfRecord: PRESERVED_SPECIMEN

##### Distribution

Turkey (Kolarov and Gürbüz 2010).

##### Diagnosis

Fig. 1

#### Banchinae

Wesmael, 1845

66239039-725C-5F6A-828D-74D3D84B2733

#### Lissonota deversor

Gravenhorst, 1829

D5506CD4-FC41-54F9-BC02-E1390009205D

https://www.gbif.org/occurrence/5084992953

##### Materials

- **Type status:** Other material. **Occurrence:** occurrenceDetails: http://api.gbif.org/v1/occurrence/search?occurrenceId=2a8de3ed-45e4-4c08-9bb3-2d6f61aa5e2d; catalogNumber: NHMUK016200530; recordedBy: [Dr Srwa Karim Bandyan]; individualCount: 1; sex: Female; lifeStage: Adult; occurrenceStatus: PRESENT; otherCatalogNumbers: NHMUK:ecatalogue:11443880; occurrenceID: 5197C92B-A2BB-584C-8F8D-71ABD2E28182; **Taxon:** higherClassification: Animalia; Arthropoda; Insecta; Hymenoptera; Ichneumonoidea; Ichneumonidae; Banchinae; taxonRank: SPECIES; **Location:** higherGeography: Asia; Iraq; Arbil; locality: Kurdistan, Qala Snigi Khwaru; decimalLatitude: 30.335; decimalLongitude: 44.327; geodeticDatum: WGS84; **Identification:** identifiedBy: Sir Anthony C. Galsworthy; **Event:** samplingProtocol: Malaise trap; eventDate: 2023-05-28; **Record Level:** institutionCode: NHMUK; collectionCode: BMNH(E); basisOfRecord: PRESERVED_SPECIMEN

##### Distribution

Very widespread in the Western Palaearctic, also in South Africa (Yu et al. 2016). This is the first record for the Middle East.

##### Diagnosis

Fig. 2

#### Campopleginae

Förster, 1869

1F8C67E8-2149-5252-B67A-6F7E0931C37C

#### Alcima orbitale

(Gravenhorst, 1829)

85797815-6E1E-5059-8F31-C7F8C4C8417E

https://www.gbif.org/occurrence/5084999924

##### Materials

- **Type status:** Other material. **Occurrence:** occurrenceDetails: http://api.gbif.org/v1/occurrence/search?occurrenceId=3d842f15-4aa1-4c79-b19b-6ca88c16245a; catalogNumber: NHMUK016200522; recordedBy: [Dr Srwa Karim Bandyan]; individualCount: 1; sex: Female; lifeStage: Adult; occurrenceStatus: PRESENT; otherCatalogNumbers: NHMUK:ecatalogue:11443852; occurrenceID: 7A315F2D-055E-5463-A2E3-06429F25787A; **Taxon:** scientificName: Alcimaorbitale (Gravenhorst, 1829); higherClassification: Animalia; Arthropoda; Insecta; Hymenoptera; Ichneumonoidea; Ichneumonidae; Campopleginae; taxonRank: SPECIES; taxonomicStatus: ACCEPTED; **Location:** higherGeography: Asia; Iraq; Arbil; locality: Kurdistan, Qala Snigi Khwaru; decimalLatitude: 30.335; decimalLongitude: 44.327; geodeticDatum: WGS84; **Identification:** identifiedBy: Sir Anthony C. Galsworthy; **Event:** samplingProtocol: Malaise trap; eventDate: 2023-05-28; **Record Level:** institutionCode: NHMUK; collectionCode: BMNH(E); basisOfRecord: PRESERVED_SPECIMEN

##### Distribution

Very widespread in the Eastern and Western Palaearctic (Yu et al. 2016).

##### Diagnosis

Fig. 3

#### Campoletis scyticus

Riedel, 2017

58393360-FCD5-562C-8D16-D5A7CE245633

https://www.gbif.org/occurrence/5084989949

##### Materials

- **Type status:** Other material. **Occurrence:** occurrenceDetails: http://api.gbif.org/v1/occurrence/search?occurrenceId=a5e7a16a-f1d6-474a-be2e-0ff167375911; catalogNumber: NHMUK016200531; recordedBy: [Dr Srwa Karim Bandyan]; individualCount: 1; sex: Male; lifeStage: Adult; occurrenceStatus: PRESENT; otherCatalogNumbers: NHMUK:ecatalogue:11443874; occurrenceID: 2A7A3E3E-6365-5773-9EC4-C977BD647773; **Taxon:** scientificName: Campoletis Förster, 1868; higherClassification: Animalia; Arthropoda; Insecta; Hymenoptera; Ichneumonoidea; Ichneumonidae; Campopleginae; taxonRank: GENUS; taxonomicStatus: ACCEPTED; **Location:** higherGeography: Asia; Iraq; Arbil; locality: Kurdistan, Qala Snigi Khwaru; decimalLatitude: 30.335; decimalLongitude: 44.327; geodeticDatum: WGS84; **Identification:** identifiedBy: Sir Anthony C. Galsworthy; **Event:** samplingProtocol: Malaise trap; eventDate: 2023-05-28; **Record Level:** institutionCode: NHMUK; collectionCode: BMNH(E); basisOfRecord: PRESERVED_SPECIMEN

##### Distribution

Afghanistan (Vas 2019), Bulgaria, Kazakhstan, Kyrgyz Republic, Morocco, Turkey, Turkmenistan (Riedel 2017).

##### Diagnosis

Fig. 4

#### Casinaria tenuiventris

(Gravenhorst, 1829)

8EF445D6-190C-50DC-9F38-52E69927FD2E

https://www.gbif.org/occurrence/5084982672

##### Materials

- **Type status:** Other material. **Occurrence:** occurrenceDetails: http://api.gbif.org/v1/occurrence/search?occurrenceId=7f3bc858-071b-451f-adf5-68deddef4195; catalogNumber: NHMUK010888455; recordedBy: [Dr Srwa Karim Bandyan']; individualCount: 1; sex: Female; lifeStage: Adult; occurrenceStatus: PRESENT; otherCatalogNumbers: NHMUK:ecatalogue:11380781; occurrenceID: C0AEF48D-DDC6-510A-9859-244923579979; **Taxon:** scientificName: Casinariatenuiventris (Gravenhorst, 1829); higherClassification: Animalia; Arthropoda; Insecta; Hymenoptera; Ichneumonoidea; Ichneumonidae; Campopleginae; taxonRank: SPECIES; taxonomicStatus: ACCEPTED; **Location:** higherGeography: Asia; Iraq; Arbil; locality: Kurdistan, Qala Snigi Khwaru; decimalLatitude: 30.335; decimalLongitude: 44.327; geodeticDatum: WGS84; **Identification:** identifiedBy: Sir Anthony C. Galsworthy; **Event:** samplingProtocol: Malaise trap; eventDate: 2023-05-28; **Record Level:** institutionCode: NHMUK; collectionCode: BMNH(E); basisOfRecord: PRESERVED_SPECIMEN

##### Distribution

Very widespread in the Eastern and Western Palaearctic; introduced into USA (Yu et al. 2016).

##### Diagnosis

Fig. 5

#### Cryptinae

Kirby, 1837

31B72B96-AD1E-5DC2-8EF5-779FFEB86FB2

#### Cryptus spinosus

Gravenhorst, 1829

87D4C9AB-0E9F-553A-B00B-8736174A5643

https://www.gbif.org/occurrence/5084987659

##### Materials

- **Type status:** Other material. **Occurrence:** occurrenceDetails: http://api.gbif.org/v1/occurrence/search?occurrenceId=26734430-224d-4959-901f-f7cf7c646f38; catalogNumber: NHMUK010888446; recordedBy: [Dr Srwa Karim Bandyan]; individualCount: 1; sex: Female; lifeStage: Adult; occurrenceStatus: PRESENT; otherCatalogNumbers: NHMUK:ecatalogue:11380771; occurrenceID: 63906DD6-8A5A-5F18-9B48-C61150708099; **Taxon:** scientificName: Cryptusspinosus Gravenhorst, 1829; higherClassification: Animalia; Arthropoda; Insecta; Hymenoptera; Ichneumonoidea; Ichneumonidae; Cryptinae; taxonRank: SPECIES; taxonomicStatus: ACCEPTED; **Location:** higherGeography: Asia; Iraq; Arbil; locality: Kurdistan, Qala Snigi Khwaru; decimalLatitude: 30.335; decimalLongitude: 44.327; geodeticDatum: WGS84; **Identification:** identifiedBy: Dr Gavin R. Broad; **Event:** samplingProtocol: Malaise trap; eventDate: 2023-05-28; **Record Level:** institutionCode: NHMUK; collectionCode: BMNH(E); basisOfRecord: PRESERVED_SPECIMEN

##### Distribution

Very widespread in the Western Palaearctic; also recorded from Central Asia and North Africa (Yu et al. 2016).

##### Diagnosis

Fig. 6

#### Ctenopelmatinae

Förster, 1869

9430600B-CEDD-57E2-9ABF-0B836A0B5590

#### Perilissus dissimilator

Aubert, 1987

6FC2FE14-68F5-5960-95FC-A2888A62E690

https://www.gbif.org/occurrence/5084988700

##### Materials

- **Type status:** Other material. **Occurrence:** occurrenceDetails: http://api.gbif.org/v1/occurrence/search?occurrenceId=d6649fc8-3972-4bd9-b830-666bc20c3eae; catalogNumber: NHMUK010888504; recordedBy: [Dr Srwa Karim Bandyan']; individualCount: 1; sex: Female; lifeStage: Adult; occurrenceStatus: PRESENT; otherCatalogNumbers: NHMUK:ecatalogue:11381241; occurrenceID: 3048ED26-7B77-5C5F-A42C-09421EC7AD71; **Taxon:** scientificName: Perilissus dissimilitor Aubert, 1987; higherClassification: Animalia; Arthropoda; Insecta; Hymenoptera; Ichneumonoidea; Ichneumonidae; Ctenopelmatinae; taxonRank: SPECIES; taxonomicStatus: ACCEPTED; **Location:** higherGeography: Asia; Iraq; Arbil; locality: Kurdistan, Qala Snigi Khwaru; decimalLatitude: 30.335; decimalLongitude: 44.327; geodeticDatum: WGS84; **Identification:** identifiedBy: Dr Gavin R. Broad; **Event:** samplingProtocol: Malaise trap; eventDate: 2023-05-28; **Record Level:** institutionCode: NHMUK; collectionCode: BMNH(E); basisOfRecord: PRESERVED_SPECIMEN

##### Distribution

Austria, France, Turkey, Ukraine, former Yugoslavia (Yu et al. 2016).

##### Diagnosis

Fig. 7

#### Ichneumoninae

Letreille, 1802

F4F1B302-8939-520E-AB26-3333BF3F32A1

#### Diadromus collaris

(Gravenhorst, 1829)

3B8B612D-DCAE-5F1E-9CE9-93E793E03758

https://www.gbif.org/occurrence/5084981691

##### Materials

- **Type status:** Other material. **Occurrence:** occurrenceDetails: http://api.gbif.org/v1/occurrence/search?occurrenceId=316069ca-e822-4816-a853-f1bd4da578dc; catalogNumber: NHMUK010888459; recordedBy: [Dr Srwa Karim Bandyan]; individualCount: 1; sex: Male; lifeStage: Adult; occurrenceStatus: PRESENT; otherCatalogNumbers: NHMUK:ecatalogue:11380786; occurrenceID: D7D2CA80-EC1A-530E-8C44-4D36250ED0FD; **Taxon:** scientificName: Diadromuscollaris (Gravenhorst, 1829); higherClassification: Animalia; Arthropoda; Insecta; Hymenoptera; Ichneumonoidea; Ichneumonidae; Ichneumoninae; specificEpithet: collaris; taxonRank: SPECIES; taxonomicStatus: ACCEPTED; **Location:** higherGeography: Asia; Iraq; Arbil; stateProvince: Arbil; locality: Kurdistan, Qala Snigi Khwaru; decimalLatitude: 30.335; decimalLongitude: 44.327; geodeticDatum: WGS84; **Identification:** identifiedBy: Dr Gavin R. Broad; **Event:** samplingProtocol: Malaise trap; eventDate: 2023-05-28; day: 28; **Record Level:** institutionCode: NHMUK; collectionCode: BMNH(E); basisOfRecord: PRESERVED_SPECIMEN

##### Distribution

Very widespread in the Eastern and Western Palaearctic; also Australia, New Zealand, South Africa, Mexico (Yu et al. 2016).

##### Diagnosis

Fig. 8

#### Pimplinae

Wesmael, 1845

039AF8D0-31EC-5C60-8E0D-8E8FCD86D86C

#### Clistopyga incitator

(Fabricius, 1793)

33D74967-B718-51A6-A91B-3349D9B3C119

https://www.gbif.org/occurrence/5084977706

##### Materials

- **Type status:** Other material. **Occurrence:** occurrenceDetails: http://api.gbif.org/v1/occurrence/search?occurrenceId=6e484e1f-93b1-4911-869f-ea43a5470296; catalogNumber: NHMUK010888497; recordedBy: [Dr Srwa Karim Bandyan']; individualCount: 1; sex: Female; lifeStage: Adult; occurrenceStatus: PRESENT; otherCatalogNumbers: NHMUK:ecatalogue:11381222; occurrenceID: 21DBB97C-B501-5248-BF16-069797BAEA76; **Taxon:** scientificName: Clistopygaincitator (Fabricius, 1793); higherClassification: Animalia; Arthropoda; Insecta; Hymenoptera; Ichneumonoidea; Ichneumonidae; Pimplinae; taxonRank: SPECIES; taxonomicStatus: ACCEPTED; **Location:** higherGeography: Asia; Iraq; Arbil; locality: Kurdistan, Qala Snigi Khwaru; decimalLatitude: 30.335; decimalLongitude: 44.327; geodeticDatum: WGS84; **Identification:** identifiedBy: Dr Gavin R. Broad; **Event:** samplingProtocol: Malaise trap; eventDate: 2023-05-28; **Record Level:** institutionCode: NHMUK; collectionCode: BMNH(E); basisOfRecord: PRESERVED_SPECIMEN

##### Distribution

Western Palaearctic, Kenya and South Korea (Yu et al. 2016).

##### Diagnosis

Fig. 9

#### Itoplectis tunetana

(Schmiedeknecht, 1914)

03427702-59EB-5F16-8F4A-A0D4DF80ADD4

https://www.gbif.org/occurrence/5084978664

##### Materials

- **Type status:** Other material. **Occurrence:** occurrenceDetails: http://api.gbif.org/v1/occurrence/search?occurrenceId=321b79a5-f421-4282-b07f-16abbfce449f; catalogNumber: NHMUK010888501; recordedBy: [Dr Srwa Karim Bandyan]; individualCount: 1; sex: Male; lifeStage: Adult; occurrenceStatus: PRESENT; otherCatalogNumbers: NHMUK:ecatalogue:11381236; occurrenceID: BDCC4B49-C679-5D79-8CF0-ECFD7451EE7C; **Taxon:** scientificName: Itoplectistunetana (Schmiedeknecht, 1914); higherClassification: Animalia; Arthropoda; Insecta; Hymenoptera; Ichneumonoidea; Ichneumonidae; Pimplinae; taxonRank: SPECIES; taxonomicStatus: ACCEPTED; **Location:** higherGeography: Asia; Iraq; Arbil; stateProvince: Arbil; locality: Kurdistan, Qala Snigi Khwaru; decimalLatitude: 30.335; decimalLongitude: 44.327; geodeticDatum: WGS84; **Identification:** identifiedBy: Dr Gavin R. Broad; **Event:** samplingProtocol: Malaise trap; eventDate: 2023-05-28; **Record Level:** institutionCode: NHMUK; collectionCode: BMNH(E); basisOfRecord: PRESERVED_SPECIMEN

##### Distribution

Very widespread in the Eastern and Western Palaearctic (Yu et al. 2016).

##### Diagnosis

Fig. 10

#### Pimpla flavicoxis

Thomson, 1877

0E380E14-AFF4-5E73-8D15-F4DAA2BE4965

https://www.gbif.org/occurrence/5084975663

##### Materials

- **Type status:** Other material. **Occurrence:** occurrenceDetails: http://api.gbif.org/v1/occurrence/search?occurrenceId=9d3993ed-9334-4553-8441-10674a2675b4; catalogNumber: NHMUK010888502; recordedBy: [Dr Srwa Karim Bandyan]; individualCount: 1; sex: Male; lifeStage: Adult; occurrenceStatus: PRESENT; otherCatalogNumbers: NHMUK:ecatalogue:11381237; occurrenceID: 732145A3-15D5-5C96-8C0F-4504332EB3EE; **Taxon:** scientificName: Pimplaflavicoxis Thomson, 1877; higherClassification: Animalia; Arthropoda; Insecta; Hymenoptera; Ichneumonoidea; Ichneumonidae; Pimplinae; taxonRank: SPECIES; taxonomicStatus: ACCEPTED; **Location:** higherGeography: Asia; Iraq; Arbil; locality: Kurdistan, Qala Snigi Khwaru; decimalLatitude: 30.335; decimalLongitude: 44.327; geodeticDatum: WGS84; **Identification:** identifiedBy: Dr Gavin R. Broad; **Event:** samplingProtocol: Malaise trap; eventDate: 2023-05-28; **Record Level:** institutionCode: NHMUK; collectionCode: BMNH(E); basisOfRecord: PRESERVED_SPECIMEN

##### Distribution

Western Palaearctic, Eastern Russia (Irkutsk, Kemerovo) (Yu et al. 2016).

##### Diagnosis

Fig. 11

#### Pimpla spuria

Gravenhorst, 1829

C4727C29-4FFE-5FBE-ADCF-4AAEFE2DA71F

https://www.gbif.org/occurrence/5084981719

##### Materials

- **Type status:** Other material. **Occurrence:** occurrenceDetails: http://api.gbif.org/v1/occurrence/search?occurrenceId=a538755a-21fa-42ba-9105-9de545b4f05d; catalogNumber: NHMUK010888498; recordedBy: [Dr Srwa Karim Bandyan]; individualCount: 1; sex: Female; lifeStage: Adult; occurrenceStatus: PRESENT; otherCatalogNumbers: NHMUK:ecatalogue:11381230; occurrenceID: C97B9B64-8A9A-5CFF-9BBC-3310C300A573; **Taxon:** scientificName: Pimplaspuria Gravenhorst, 1829; higherClassification: Animalia; Arthropoda; Insecta; Hymenoptera; Ichneumonoidea; Ichneumonidae; Pimplinae; taxonRank: SPECIES; taxonomicStatus: ACCEPTED; **Location:** higherGeography: Asia; Iraq; Arbil; locality: Kurdistan, Qala Snigi Khwaru; decimalLatitude: 30.335; decimalLongitude: 44.327; geodeticDatum: WGS84; **Identification:** identifiedBy: Dr Gavin R. Broad; **Event:** samplingProtocol: Malaise trap; eventDate: 2023-05-28; **Record Level:** institutionCode: NHMUK; collectionCode: BMNH(E); basisOfRecord: PRESERVED_SPECIMEN

##### Distribution

Very widespread in the Eastern and Western Palaearctic; North Africa (Yu et al. 2016).

##### Diagnosis

Fig. 12

#### Zaglyptus multicolor

(Gravenhorst, 1829)

A9197368-E294-57ED-9E78-5E2F369E427A

https://www.gbif.org/occurrence/5084977707

##### Materials

- **Type status:** Other material. **Occurrence:** occurrenceDetails: http://api.gbif.org/v1/occurrence/search?occurrenceId=ceecf3c4-64a7-469c-849d-d8891d73448a; catalogNumber: NHMUK010888499; recordedBy: [Dr Srwa Karim Bandyan]; individualCount: 1; sex: Male; lifeStage: Adult; occurrenceStatus: PRESENT; otherCatalogNumbers: NHMUK:ecatalogue:11381231; occurrenceID: 2E7BBFD4-AD6C-5397-AA2D-9D59BB365264; **Taxon:** scientificName: Zaglyptusmulticolor (Gravenhorst, 1829); higherClassification: Animalia; Arthropoda; Insecta; Hymenoptera; Ichneumonoidea; Ichneumonidae; Pimplinae; taxonRank: SPECIES; taxonomicStatus: ACCEPTED; **Location:** higherGeography: Asia; Iraq; Arbil; continent: ASIA; locality: Kurdistan, Qala Snigi Khwaru; decimalLatitude: 30.335; decimalLongitude: 44.327; geodeticDatum: WGS84; **Identification:** identifiedBy: Dr Gavin R. Broad; **Event:** samplingProtocol: Malaise trap; eventDate: 2023-05-28; **Record Level:** institutionCode: NHMUK; collectionCode: BMNH(E); basisOfRecord: PRESERVED_SPECIMEN

##### Distribution

Very widespread in the Eastern and Western Palaearctic (Yu et al. 2016).

##### Diagnosis

Fig. 13

#### Zatypota bohemani

(Holmgren, 1860)

318AA9D7-0695-5522-9ECB-A8730F2672DE

https://www.gbif.org/occurrence/5084985673

##### Materials

- **Type status:** Other material. **Occurrence:** occurrenceDetails: http://api.gbif.org/v1/occurrence/search?occurrenceId=b255abd5-2d55-4f96-ae66-dabb021a3618; catalogNumber: NHMUK010888500; recordedBy: [Dr Srwa Karim Bandyan]; individualCount: 1; sex: Female; lifeStage: Adult; occurrenceStatus: PRESENT; otherCatalogNumbers: NHMUK:ecatalogue:11381234; occurrenceID: 29F67328-081A-5441-9989-974C53C7A9AA; **Taxon:** scientificName: Zatypotabohemani (Holmgren, 1860); higherClassification: Animalia; Arthropoda; Insecta; Hymenoptera; Ichneumonoidea; Ichneumonidae; Pimplinae; taxonRank: SPECIES; taxonomicStatus: ACCEPTED; **Location:** higherGeography: Asia; Iraq; Arbil; locality: Kurdistan, Qala Snigi Khwaru; decimalLatitude: 30.335; decimalLongitude: 44.327; geodeticDatum: WGS84; **Identification:** identifiedBy: Dr Gavin R. Broad; **Event:** samplingProtocol: Malaise trap; eventDate: 2023-05-28; **Record Level:** institutionCode: NHMUK; collectionCode: BMNH(E); basisOfRecord: PRESERVED_SPECIMEN

##### Distribution

Very widespread in the Western Palaearctic; also Iran, Kazakhstan, Lebanon, Mongolia, Tadjikistan, Canada (British Columbia), USA (Montana) (Yu et al. 2016).

##### Diagnosis

Fig. 14

## Supplementary Material

XML Treatment for Acaenitinae

XML Treatment for Phaenolobus hilalii

XML Treatment for Banchinae

XML Treatment for Lissonota deversor

XML Treatment for Campopleginae

XML Treatment for Alcima orbitale

XML Treatment for Campoletis scyticus

XML Treatment for Casinaria tenuiventris

XML Treatment for Cryptinae

XML Treatment for Cryptus spinosus

XML Treatment for Ctenopelmatinae

XML Treatment for Perilissus dissimilator

XML Treatment for Ichneumoninae

XML Treatment for Diadromus collaris

XML Treatment for Pimplinae

XML Treatment for Clistopyga incitator

XML Treatment for Itoplectis tunetana

XML Treatment for Pimpla flavicoxis

XML Treatment for Pimpla spuria

XML Treatment for Zaglyptus multicolor

XML Treatment for Zatypota bohemani

## Figures and Tables

**Figure 1.**
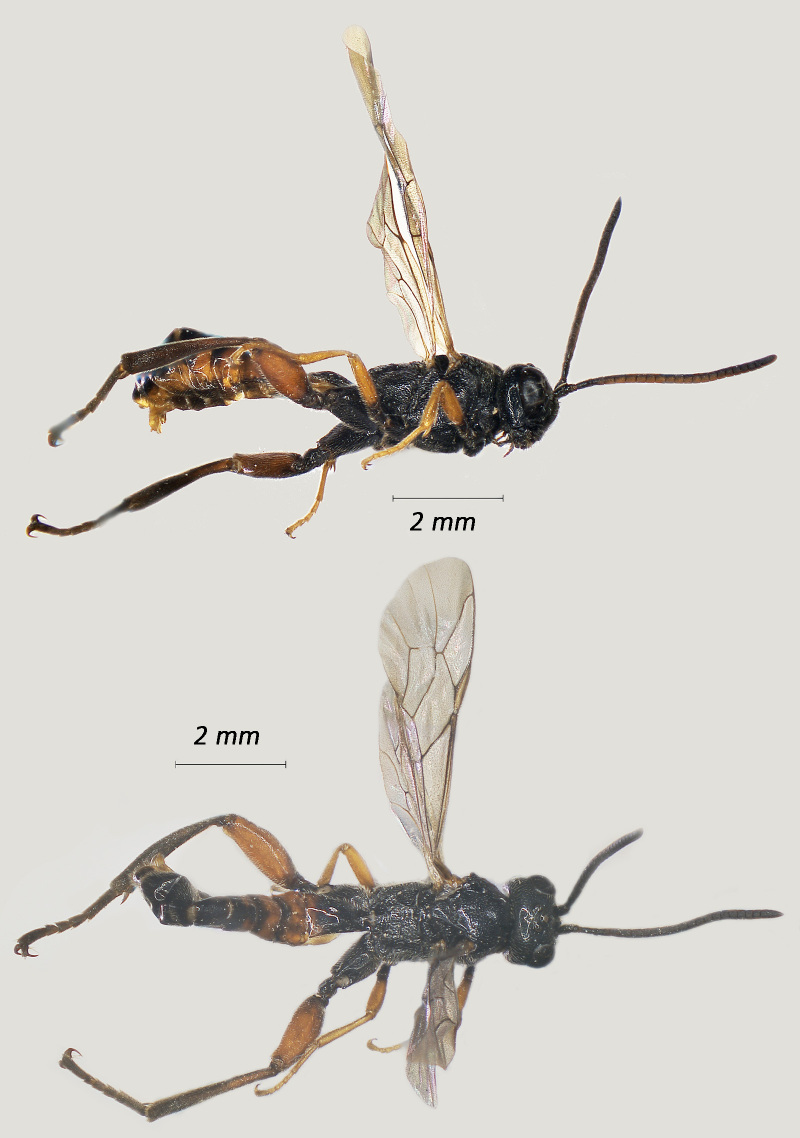
*Phaenolobushilalii*, ♂ Adult. Lateral view (above) and dorsal view (below).

**Figure 2.**
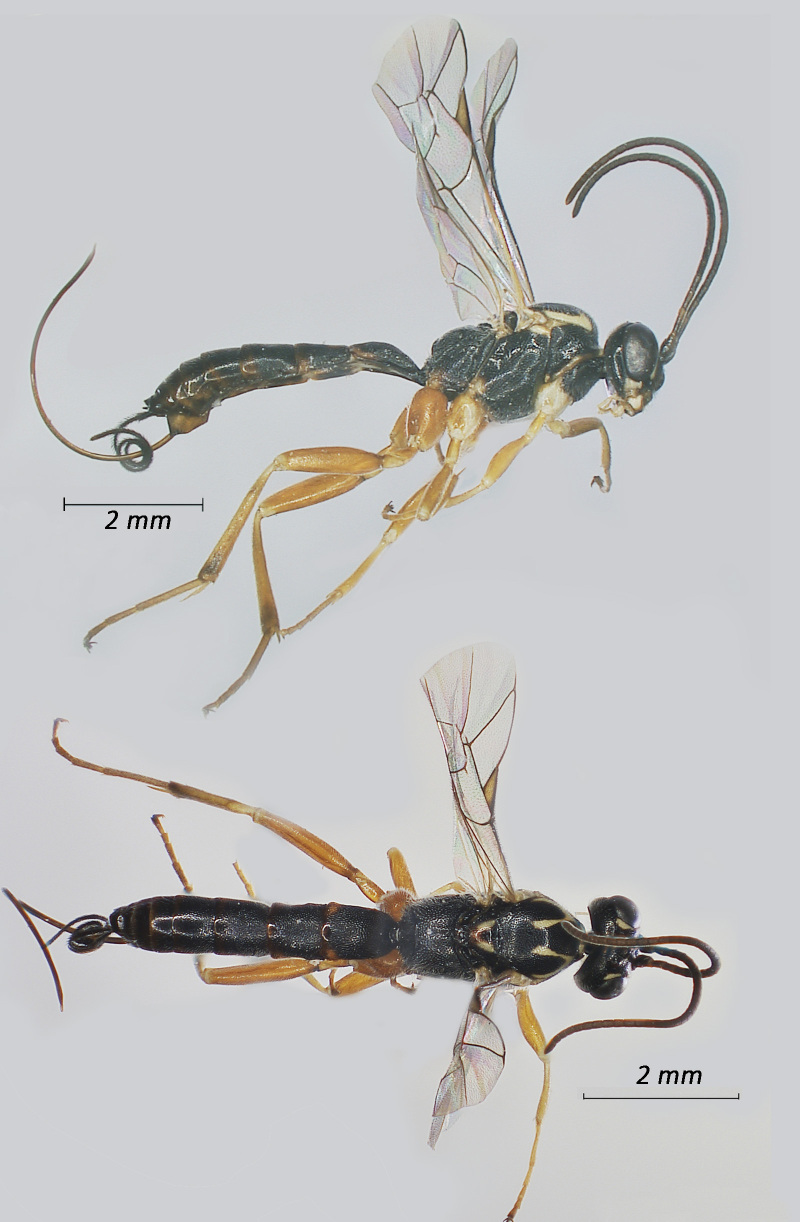
*Lissonotadeversor*, ♀ Adult. Lateral view (above) and dorsal view (below)

**Figure 3.**
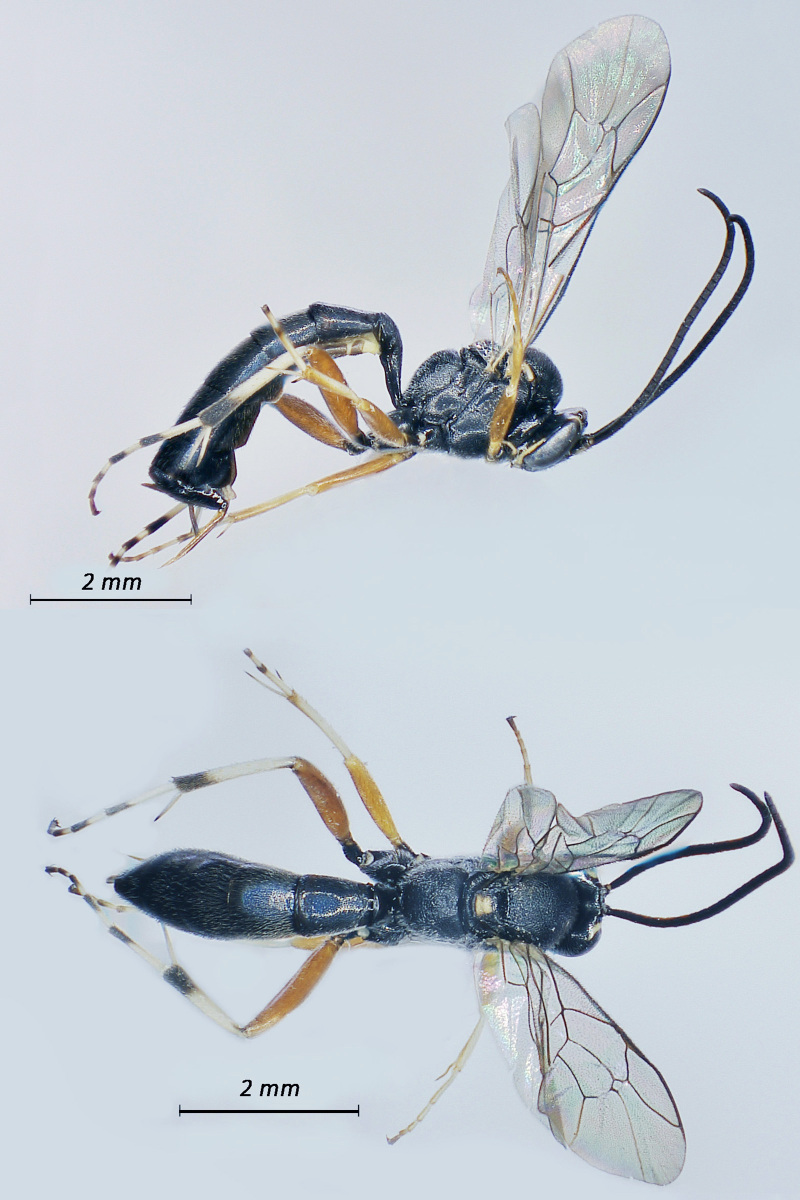
*Alcimaorbitale*, ♀Adult. Lateral view (above) and dorsal view (below).

**Figure 4.**
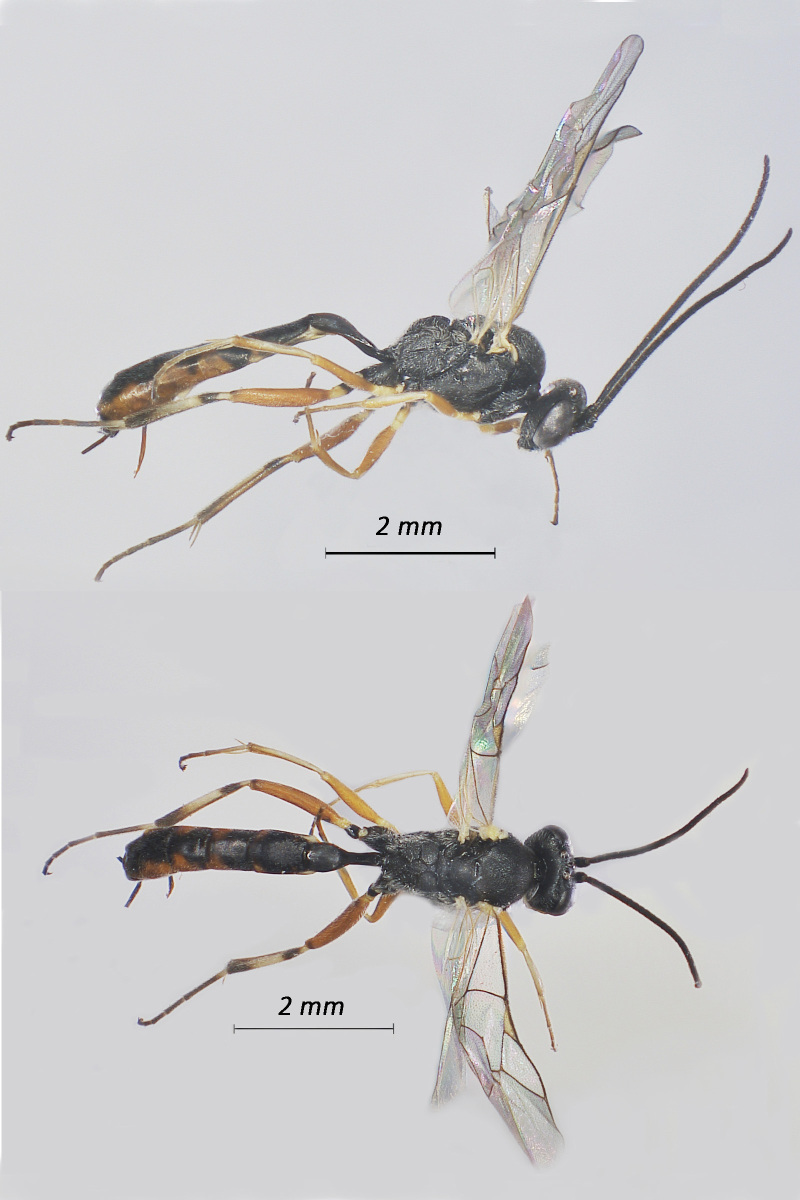
*Campoletisscyticus*, ♂Adult. Lateral view (above) and dorsal view (below).

**Figure 5.**
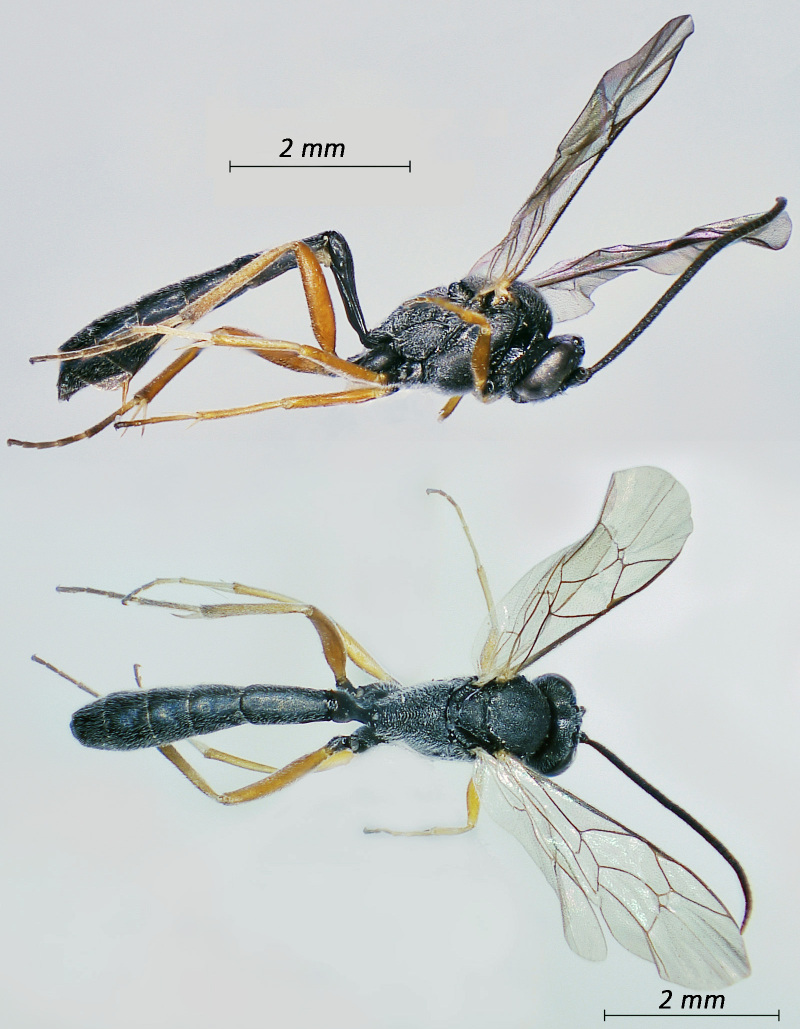
*Casinariatenuiventris*, ♀Adult. Lateral view (above) and dorsal view (below).

**Figure 6.**
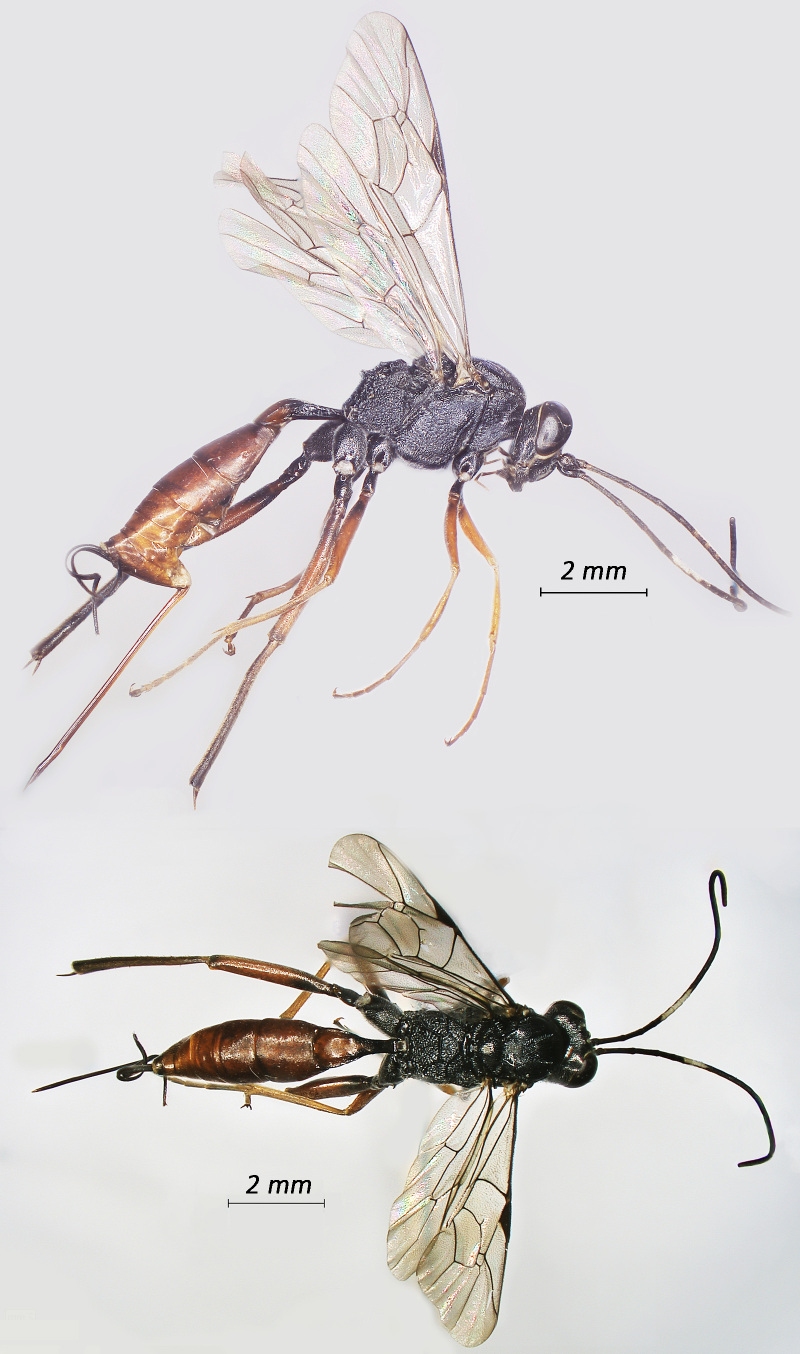
*Cryptusspinosus*, ♀ Adult. Lateral view (above) and dorsal view (below).

**Figure 7.**
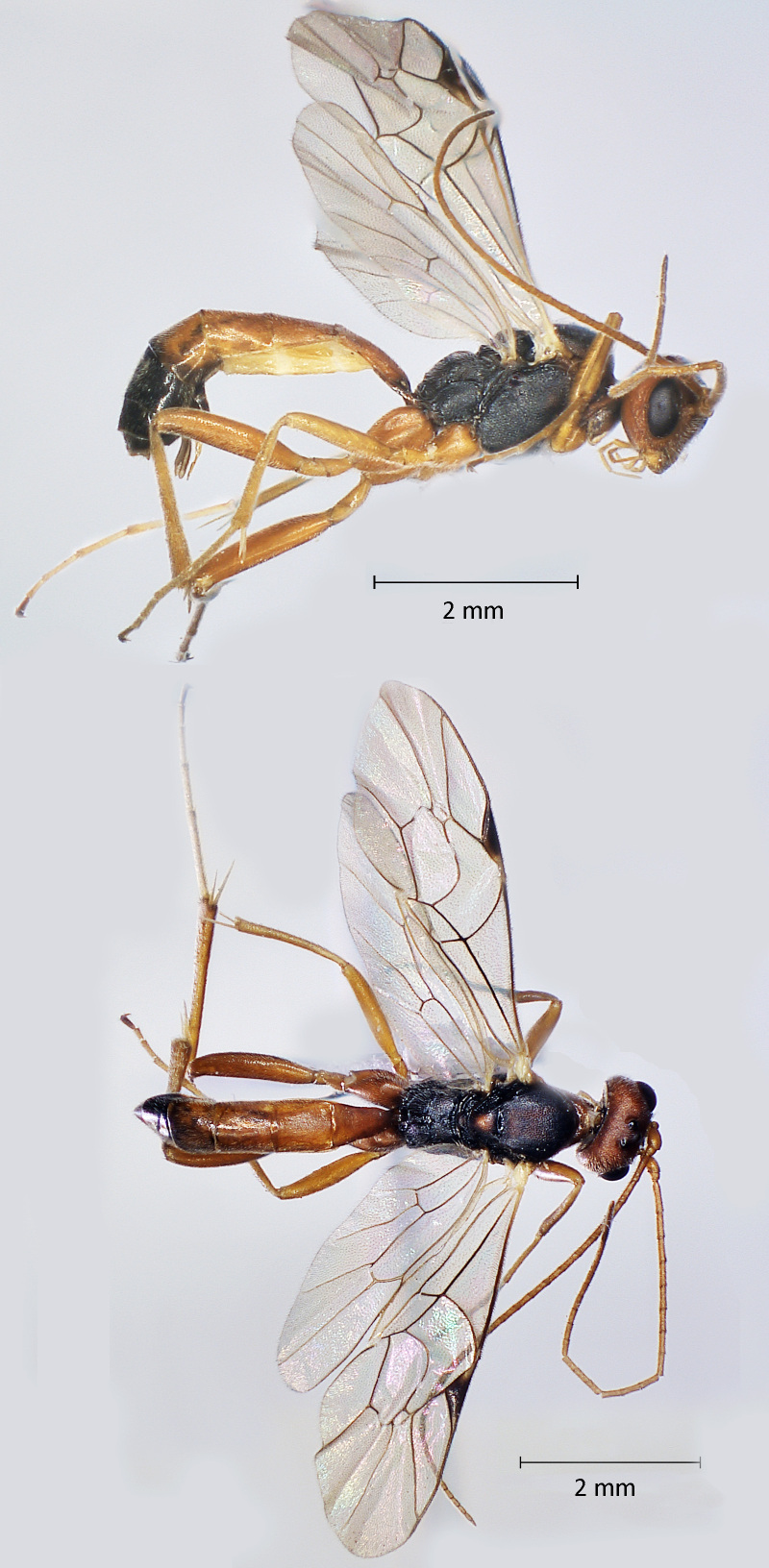
*Perilissusdissimilator*, ♀ Adult. Lateral view (above) and dorsal view (below).

**Figure 8.**
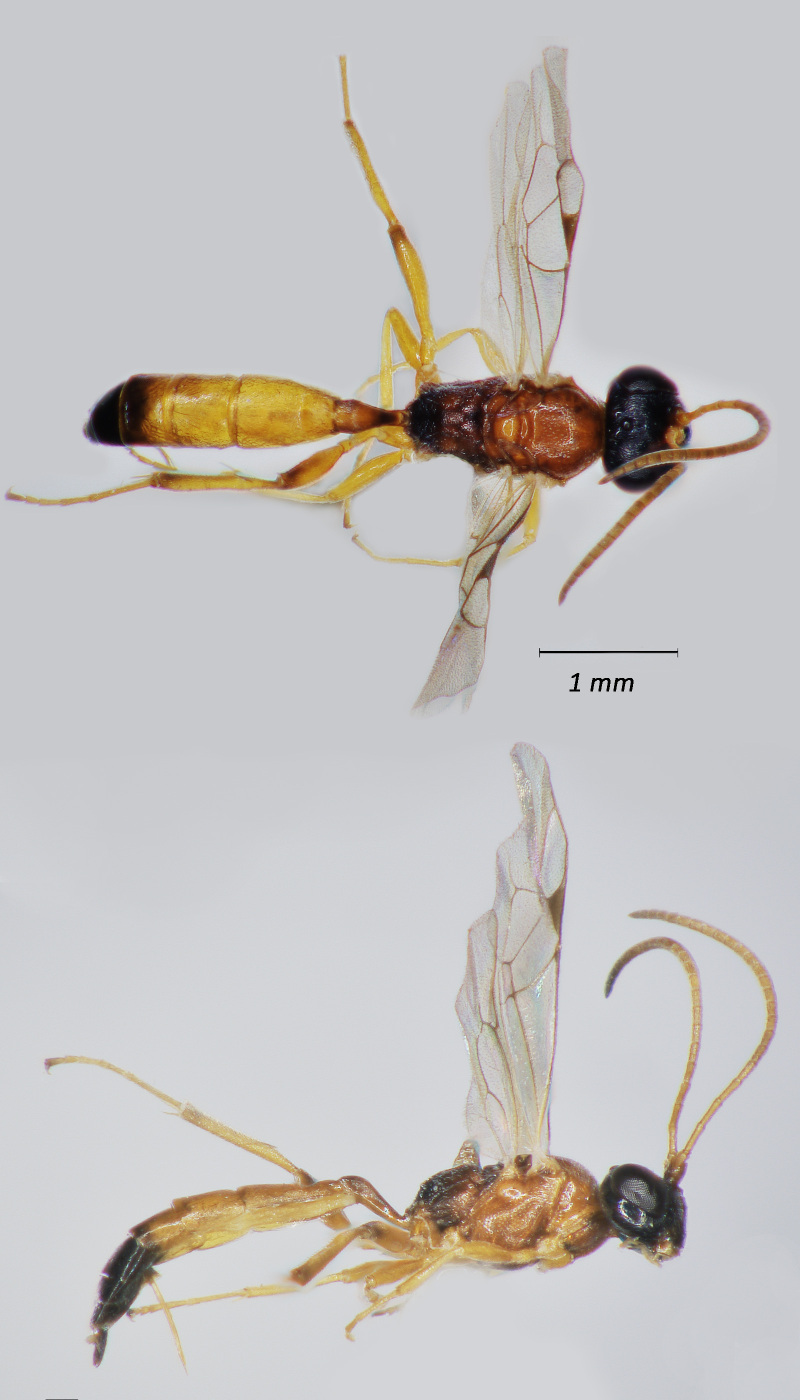
*Diadromuscollaris*, ♂ Adult. Lateral view (above) and dorsal view (below).

**Figure 9.**
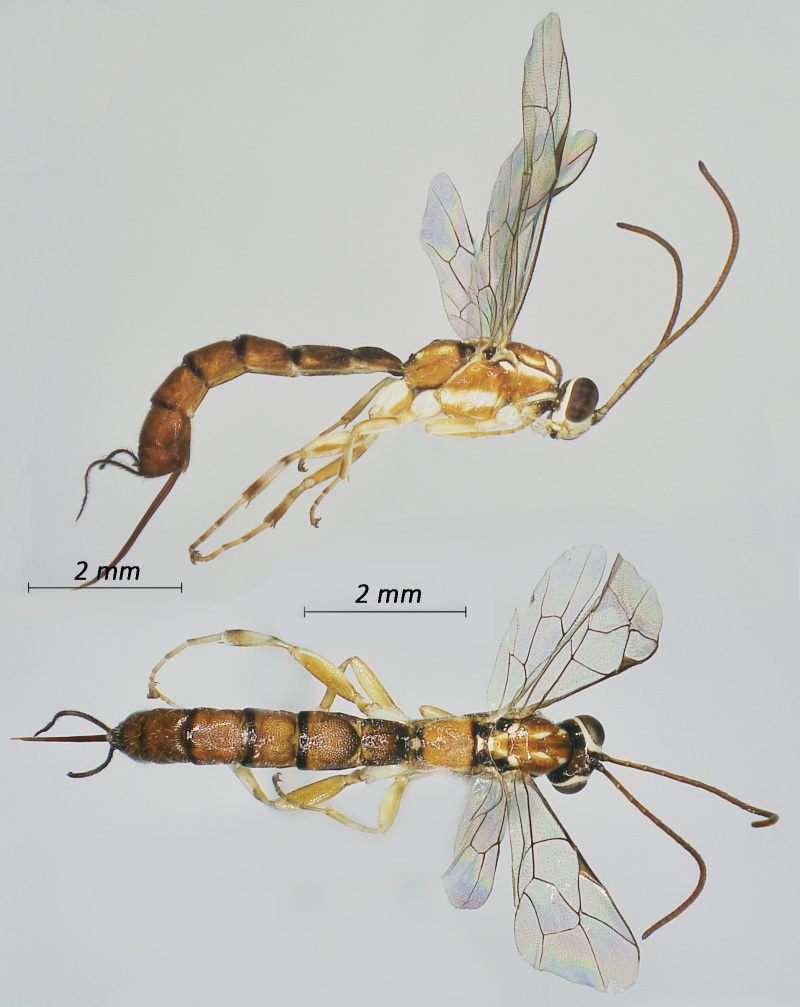
*Clistopygaincitator*, ♀ Adult. Lateral view (above) and dorsal view (below).

**Figure 10.**
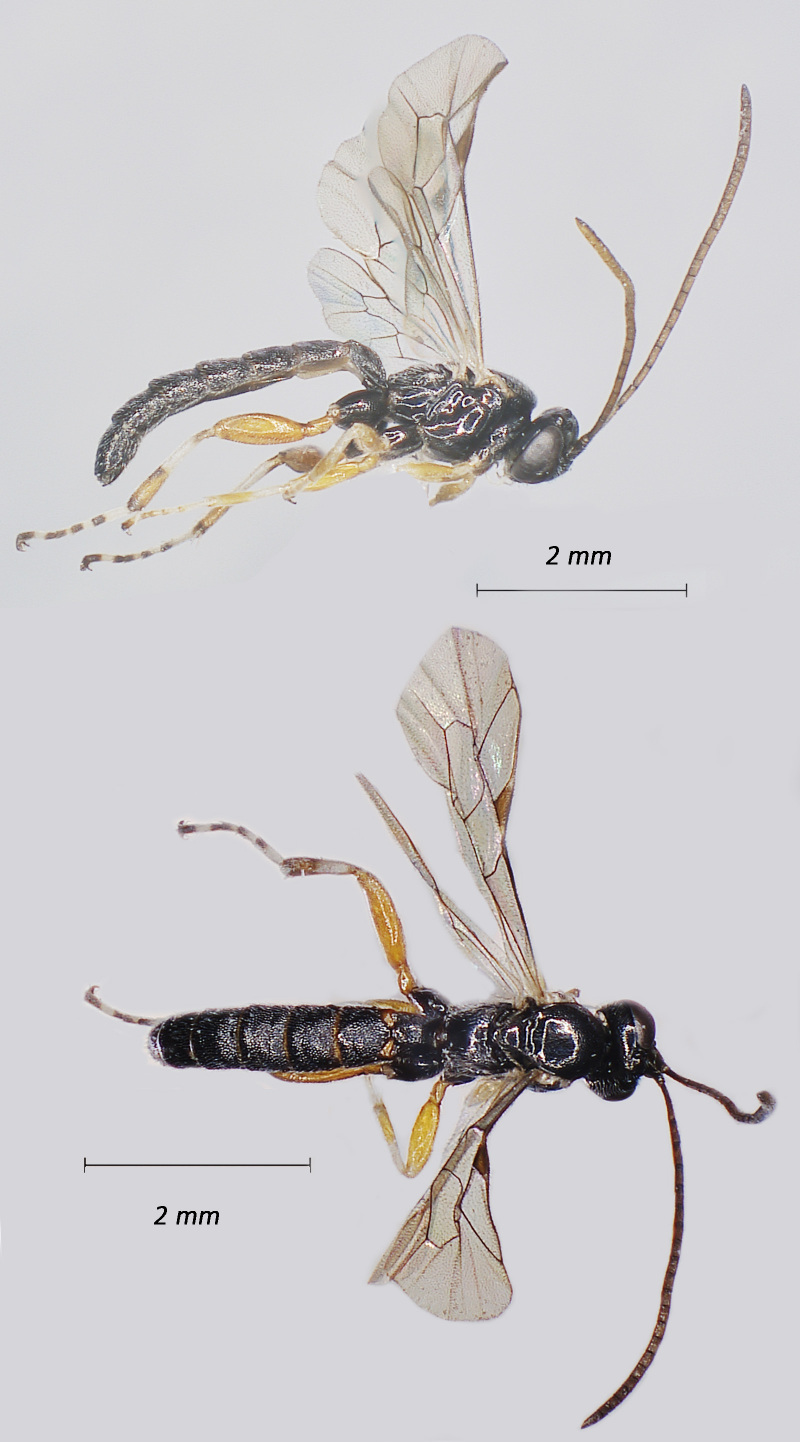
*Itoplectistunetana*, ♂ Adult. Lateral view (above) and dorsal view (below).

**Figure 11.**
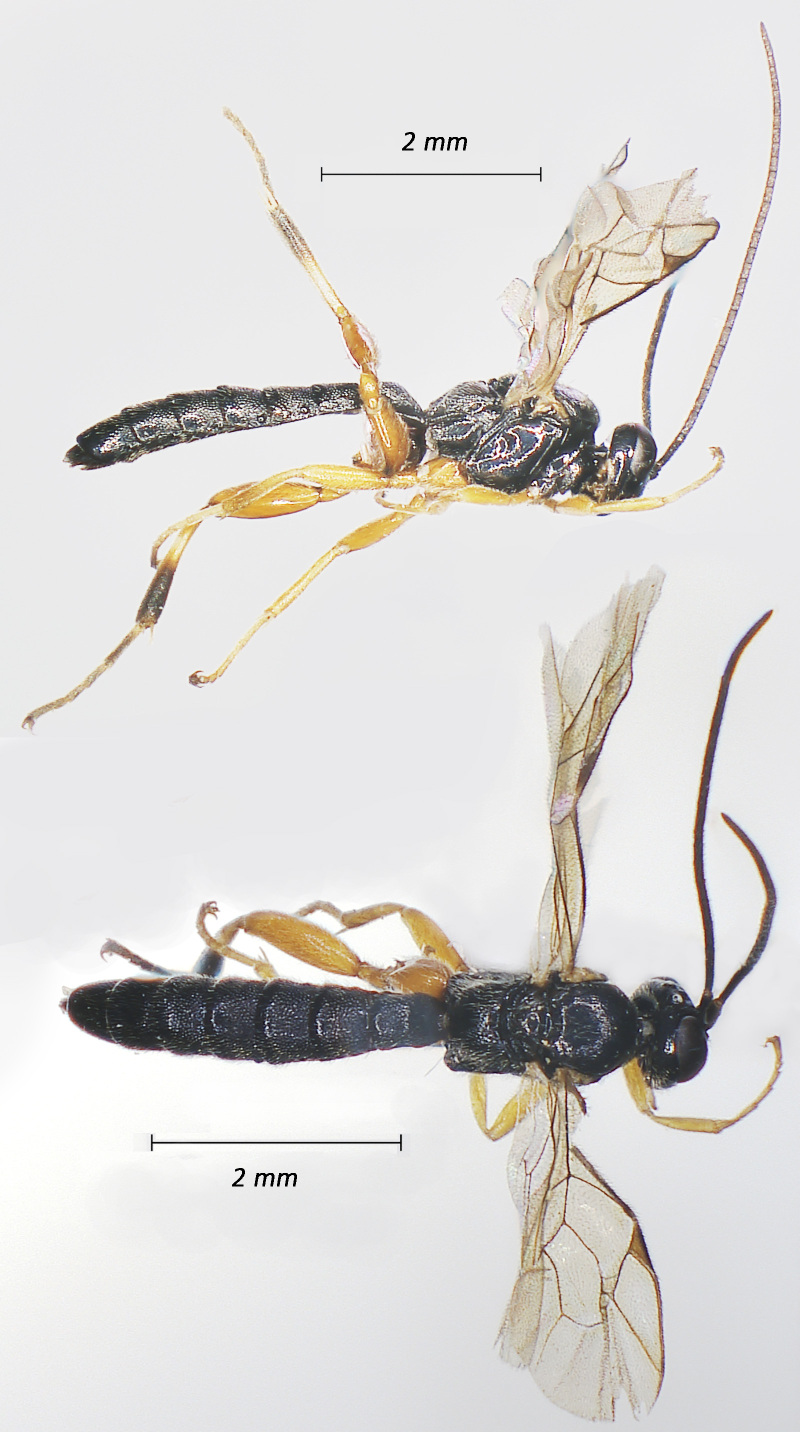
*Pimplaflavicoxis*, ♂ Adult. Lateral view (above) and dorsal view (below).

**Figure 12.**
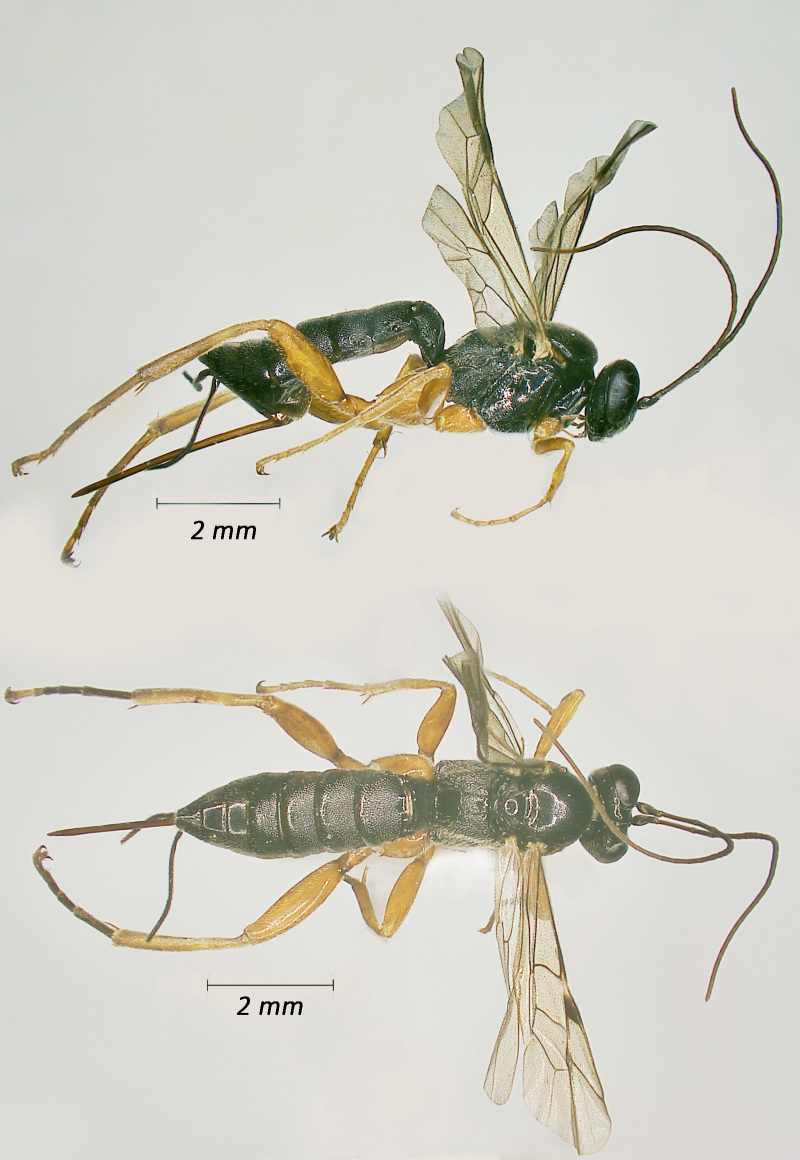
*Pimplaspuria*, ♀ Adult. Lateral view (above) and dorsal view (below).

**Figure 13.**
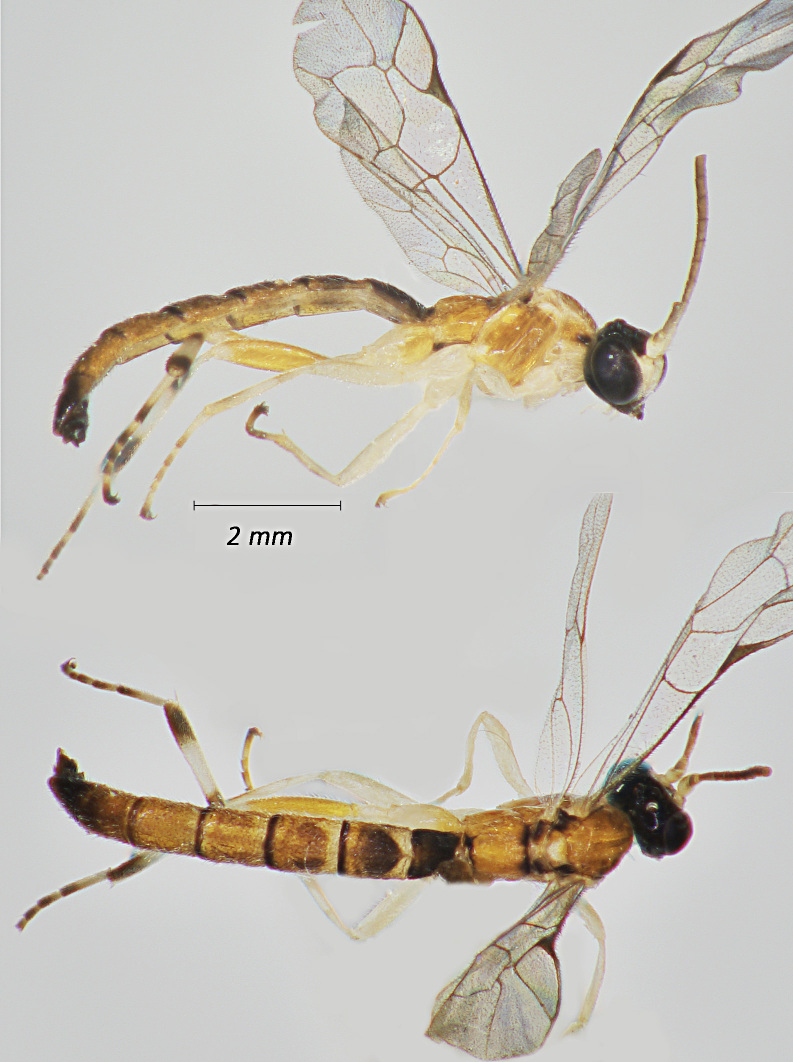
*Zaglyptusmulticolor*, ♂ Adult. Lateral view (above) and dorsal view (below).

**Figure 14.**
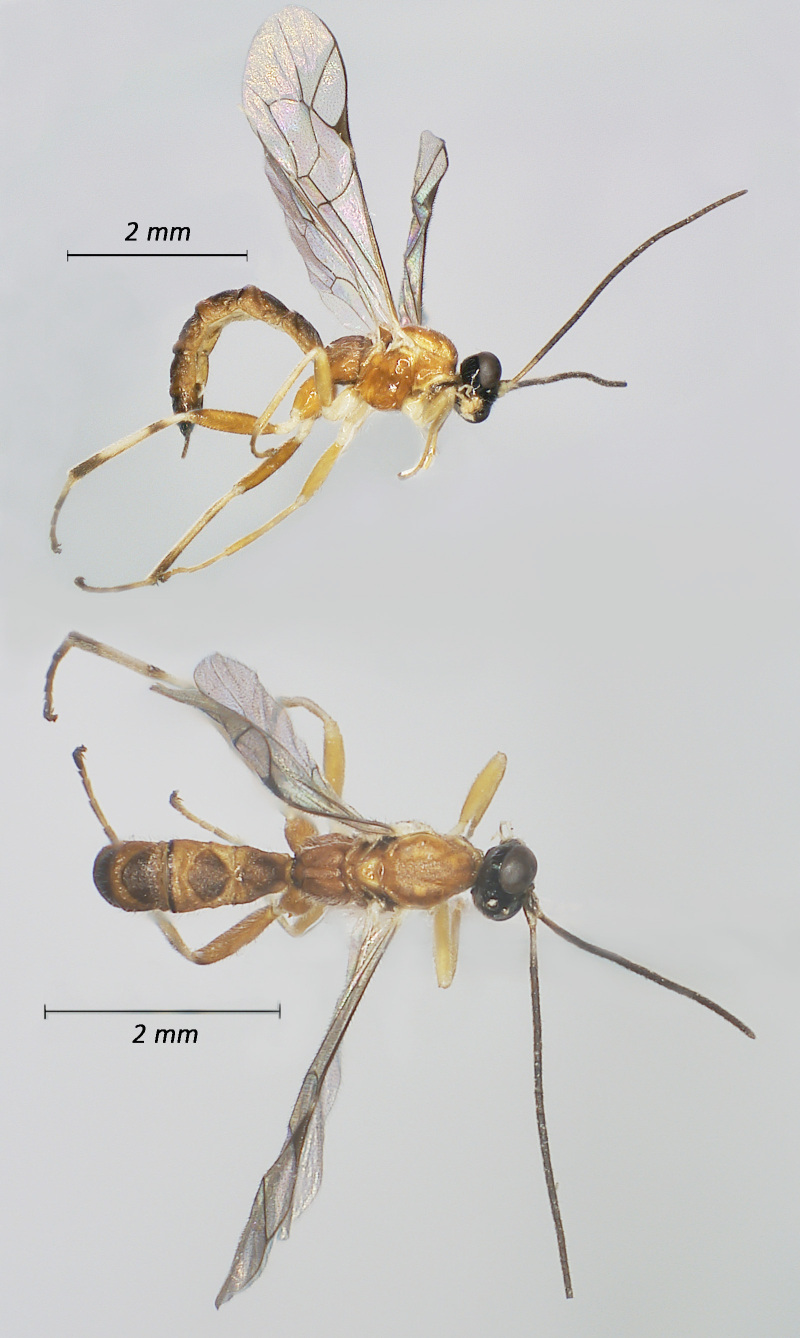
*Zatypotabohemani*, ♀ Adult. Lateral view (above) and dorsal view (below).
